# Molecular and Immune Biomarkers for Cutaneous Melanoma: Current Status and Future Prospects

**DOI:** 10.3390/cancers12113456

**Published:** 2020-11-20

**Authors:** Lorenzo Pilla, Andrea Alberti, Pierluigi Di Mauro, Maria Gemelli, Viola Cogliati, Marina Elena Cazzaniga, Paolo Bidoli, Cristina Maccalli

**Affiliations:** 1 Division of Medical Oncology, San Gerardo Hospital, University of Milano-Bicocca School of Medicine, 20900 Monza, Italy; p.dimauro001@unibs.it (P.D.M.); maria.gemelli@asst-monza.it (M.G.); viola.cogliati@unimi.it (V.C.); marina.cazzaniga@asst-monza.it (M.E.C.); p.bidoli@asst-monza.it (P.B.); 2 Medical Oncology Unit, Department of Medical and Surgical Specialties, Radiological Health Science and Public Health, University of Brescia, ASST Ospedali Civili, 25123 Brescia, Italy; a.alberti015@unibs.it; 3 Laboratory of Immune and Biological Therapy, Research Department, Sidra Medicine, Doha 26999, Qatar; cmaccalli@sidra.org

**Keywords:** biomarkers, melanoma, checkpoint inhibitor, PD-1, target therapy

## Abstract

**Simple Summary:**

The prognosis and treatment of metastatic melanoma have changed substantially since the advent of target therapy and immune checkpoint inhibitors. Thus, strategies must be developed to identify responder patients, reduce toxicities, and investigate target and immune based therapy ideal sequencing. To this aim, the determinants driving response, resistance, and adverse events, should be defined. In addition, novel oncogenic drivers should be discovered to provide new therapeutic targets. Current methods of detection, prognosis and monitoring of melanoma are based on clinical, morphological and histopathologic characteristics of the tumor. This review provides an update on prognostic and predictive biomarkers with a potential application in melanoma patients’ clinical management.

**Abstract:**

Advances in the genomic, molecular and immunological make-up of melanoma allowed the development of novel targeted therapy and of immunotherapy, leading to changes in the paradigm of therapeutic interventions and improvement of patients’ overall survival. Nevertheless, the mechanisms regulating either the responsiveness or the resistance of melanoma patients to therapies are still mostly unknown. The development of either the combinations or of the sequential treatment of different agents has been investigated but without a strongly molecularly motivated rationale. The need for robust biomarkers to predict patients’ responsiveness to defined therapies and for their stratification is still unmet. Progress in immunological assays and genomic techniques as long as improvement in designing and performing studies monitoring the expression of these markers along with the evolution of the disease allowed to identify candidate biomarkers. However, none of them achieved a definitive role in predicting patients’ clinical outcomes. Along this line, the cross-talk of melanoma cells with tumor microenvironment plays an important role in the evolution of the disease and needs to be considered in light of the role of predictive biomarkers. The overview of the relationship between the molecular basis of melanoma and targeted therapies is provided in this review, highlighting the benefit for clinical responses and the limitations. Moreover, the role of different candidate biomarkers is described together with the technical approaches for their identification. The provided evidence shows that progress has been achieved in understanding the molecular basis of melanoma and in designing advanced therapeutic strategies. Nevertheless, the molecular determinants of melanoma and their role as biomarkers predicting patients’ responsiveness to therapies warrant further investigation with the vision of developing more effective precision medicine.

## 1. Introduction

Malignant melanoma is the 5th most common cancer and represents 1.5% of all cancer deaths. However, during the last 20 years, the incidence was increased, with more than 9000 melanoma-related deaths registered in 2018 [1].

The prognosis of melanoma, particularly in the metastatic setting, over the last ten years was significantly improved [2]. This progress is mainly due to the clinical approval of target therapy with BRAF and MEK inhibitors and of immunotherapy with immune checkpoint inhibitors (ICIs), specifically those targeting the Cytotoxic T-Lymphocyte Associated Protein 4 (CTLA-4) and Programmed Death 1 (PD-1)/Ligand-1 (PD-L1) pathways. Different studies demonstrated an enhanced clinical benefit from these treatments in specific patient populations, such as early-stage patients and stage IV patients with limited tumor burden [3,4].

This suggests that in the upcoming years, it will be crucial to identify patients at higher recurrence risk and monitor for early relapse, in order to assign the best preventive approach, sparing patients at very low risk from over-treatment side effects.

A biomarker can be anything from a serum protein, detectable genetic alteration, pathology finding, or imaging finding that helps to predict the presence of disease or guide its therapeutic options. The use of biomarkers has tremendously improved predictivity of response to treatment of some specific melanoma subtypes. Indeed, genetic testing looking for BRAF mutations, has a proven role in the choice of targeted therapy. However, the identification of definitive clinically useful biomarkers for the response to ICIs of melanoma patients is still under investigation. There is no blood test that can be used for diagnosis to detect melanoma recurrence, although lactate dehydrogenase (LDH) and S-100B can be useful for monitoring.

This review examines the past, current, and future role of biomarkers in melanoma detection, treatment selection, and treatment monitoring. 

## 2. Biomarkers in Target Therapy

Melanoma is a highly heterogeneous disease from a genetic point of view. However, the identification of specific subtypes of melanoma based on distinct molecular alterations enables critical information on patient prognosis and potential treatment options [5] (Table 1). 

The identification of the mutations in the V600 codon of BRAF (35–50% of melanomas) and Q61 codons (less frequently, the G12 or G13 codon) of NRAS (10–25% of melanomas) prompted the era of target therapy [13]. 

Several next-generation sequencing (NGS) studies showed that the mutational rate of melanoma is by far higher than that reported for other solid tumors, probably as a consequence of ultraviolet radiation exposure. However, a large proportion of these mutations are bystanders, not involved in the neoplastic process [13].

Interestingly, those subtypes of melanoma in which UV does not have any involvement in the pathogenesis, display a different pattern of specific mutations. In Uveal melanoma, the most frequent driver mutation is associated with the G protein subunits. In mucosal melanoma, BRAF and NRAS mutations are far less frequent compared to cutaneous melanoma, while mutations in c-KIT are observed in 7–25% of cases [14].

The Cancer Genome Atlas Network has recently provided a potential step ahead in the comprehension of melanoma genesis, proposing a classification of melanoma based on protein, RNA and DNA analysis from over 300 melanoma patients, in addition to BRAF, NRAS, and NF1 mutational profile [15]. This genomic classification provides a step further to identify new subtypes of melanoma which can help to understand different clinical behaviors and resistance to therapy.

### 2.1. BRAF

BRAF is the most common mutated gene in cutaneous melanoma, ranging from 40–60% of cases, and leading to uncontrolled activation of the mitogen-activated protein kinases (MAPK) pathway. The two most frequent mutations of this gene are V600E and V600K [16,17], and are associated with different factors: BRAF V600E is associated with younger onset age, superficial spreading subtype and skin without chronic sun damage (CSD) (e.g., extremities), while BRAF V600K with older age, and skin with CSD (e.g., head and neck) [18,19]. The development of BRAF inhibitors such as vemurafenib, dabrafenib, and encorafenib dramatically improved the overall response rate (ORR) and the overall survival (OS) in patients with this melanoma subtype. However, despite encouraging clinical results with monotherapy [13], early development of acquired resistance through several mechanisms was noted, such as the upregulation of MEK, ERK or NRAS [20]. Combination therapy with BRAF- and MEK-inhibitors, including trametinib, cobimetinib and binimetinib, reported a prolongation in both the progression-free survival (PFS) and OS compared with single-agent BRAF inhibitors [21,22,23]. In particular, the newest doublet therapy, encorafenib/binimetinib showed even greater efficacy, improving the PFS from 7 to 15 months and the OS from 16.9 to 33.6 months [23]. 

Rare BRAF mutations (V600 non-E/K and non-V600) account for 5% of BRAF-mutated melanoma, and their role in tumorigenesis and response to target therapy is still to be elucidated. Despite most of them, such as L597V, K601E, G469A, showed BRAF and MAPK pathway activation, their prognostic and predictive role is uncertain [24]. Among these, the V600R, L597P/Q/R/S, and K601E are the most common rare BRAF mutations. 

To date, melanoma with non-V600 BRAF mutations has been mostly excluded from enrollment in clinical studies investigating the efficacy of BRAF and MEK inhibitors. A phase 2 trial, evaluating the activity and the efficacy of trametinib in patients with advanced melanoma not mutated in V600 BRAF (NCT02296112), could help to shed some light on the role of MEK inhibitors in this rare subset of melanoma.

### 2.2. NRAS

The gene N-RAS encodes for a GTPase, which plays a crucial role in the signal transduction of both the MAPK and phosphatidyl inositol 3 kinases (PI3K) pathways [25]. NRAS mutations account for 20% of cases [26] and are generally mutually exclusive with BRAF mutations, even if there is evidence of cases in which both mutations can co-exist in the same lesions [27]. However, melanoma with N-RAS mutation is frequently detected in CSD skin and associated with the nodular subtype [28,29]. The association between N-RAS alteration and worse survival for metastatic melanoma is not ultimately demonstrated, although some studies reported it to be a negative prognostic factor [26]. Several studies did not show any difference in OS; an association between NRAS mutation and poor survival or a positive correlation among the two factors were observed [26,30,31,32].

MEK-inhibition has been identified as a potential therapy in this melanoma subtype, since improved response rate and PFS were found upon treatment with binimetinib as compared to chemotherapy [6]. Nonetheless, the clinical risk/benefit ratio was not sufficient to support the approval of this compound for this indication. The combination of MEK inhibitors with PI3K, RAF, and other cell cycle inhibitors is the object of ongoing investigations [33]. 

### 2.3. KIT

Among the multiple genes affected by alterations that could potentially lead to melanoma development, mutations in c-KIT are becoming an appealing target for personalized therapy. This proto-oncogene encodes for a receptor tyrosine kinase (RTK), and its mutations were found in other cancer types [34]. Although alterations in KIT were identified in only 3% of all melanomas, they were frequently (28–39%) detected in melanoma arising from CSD skin, acral and mucosal sites [35,36]. Several trials based on targeting with kinase inhibitors c-KIT showed consistent results, obtaining an ORR of 16–29% and a median OS of 12–13 months [7,8,9,10]. Other studies investigating nilotinib, a small molecule more potent than imatinib in inhibiting KIT, showed promising clinical activity [11,37,38]. The global, single-arm, phase II TEAM trial showed 26.2% ORR (all partial responses) and OS of 18 months [11]. The kinase inhibitors dasatinib and sunitinib were also tested in this setting, with mixed results [39,40]. Superior response rates in patients harboring KIT mutations in exon 11 or 13 were noted, likely representing driver mutations responsible for melanoma growth.

### 2.4. Other Single Gene Candidate Predictive Biomarkers 

Advances in genomic sequencing technologies, coupled with the development of effective melanoma therapies, have led to the identification of genes bearing specific driver mutations and displaying the role of predictors of response. 

*NF1* gene encodes for neurofibromin, a protein that negatively regulates the MAPK pathway: it is the third most commonly mutated gene in melanoma, found in 46% of cases with BRAF and NRAS wild type, and often co-mutated with RAS-associated genes [41]. 

NF1 mutant melanoma failed to show specific clinicopathological characteristics, other than the association with advanced patients’ age [15]. 

Currently, there’s no clinical trial investigating NF-1 mutant melanoma, while a combination of MEK and PI3K or mTOR inhibitors showed promising activity in mouse models [42].

A large proportion (85–90%) of uveal melanoma harbors a driver mutation in GNAQ or GNA11: these mutations are mutually exclusive and cause an overamplification of the downstream signaling through the MAPK pathway [43,44]. Therapies specifically targeting this altered gene are not available. Treatment with selumetinib, a competitive MEK-inhibitor, failed to show any improvement in overall survival when either compared to or added to dacarbazine mono-chemotherapy [12,45]. Multiple clinical trials are ongoing to evaluate the clinical efficacy of the inhibition of mitogenic signaling mediated by GNAQ/GNA11. These include PKC inhibitors (NCT01430416, NCT01801358) and MTOR inhibitors (NCT01430416; NCT01801358.)

CDKN2A loss is found in 50% of malignant melanoma [46]. CDKN2A and CDKN2B genes block cell cycle progression through inhibition of CDK4 and retinoblastoma protein. CDK4 mutations are associated with 20% of familial melanoma [47], and it is generally associated with a worse prognosis [48,49].

Alterations of the CDK4/6 signaling pathway is observed in different types of melanoma, concomitantly with NRAS, KRAS or BRAF; thus the genetic profiling of CDK4/6 may provide insights for the targeted treatment of various types of melanoma. Preclinical studies demonstrated that CDK4/6 inhibitors could overcome the resistance to the treatment with RAS/RAF/MEK/ERK inhibitors and anti-PD-1 immunotherapy [50].

### 2.5. Resistance to Target Therapy 

The combination of BRAF inhibitors and MEK inhibitors showed clinical efficacy and long-lasting disease control in metastatic melanoma whit an OS rate of 30% at five years [22]. However, resistance mechanisms, leading to disease progression, may occur during the treatment, and about 15% of patients are refractory to the treatment [22]. Different mechanisms may be involved: genetic causes with sustained activation of the MAPK pathway, the emergence of alternative pro-oncogenic pathways, epigenetic alterations, and microenvironment modulation [51]. 

Genetic alterations of genes involved in the MAPK pathway are reported in about 50% of patients progressing to the treatment with BRAFi and MEKi; among these NRAS mutations accounted for 17% and MAP2KI-2 mutations of 15% and 8%, respectively. NRAS point mutations are detected at an early stage of treatment, usually within 12 weeks, while the presence of MAP2KI P124S and P124L in pre-treatment specimens correlated with rapid progression [52,53]. Nevertheless, genetic alterations could not be identified in 40% of patients with disease progression [54]. BRAF V600E/K amplifications have been reported in 8–13% of resistance to BRAFi and could coexist with other genetic alterations as NRAS mutations [52]. Another mechanism of escape is the onset of BRAF alternate splicing variants, which determine aberrant BRAF proteins’ dimerization and unable the BRAFi to bind it [54]. Amplifications of cMET and MITF have been associated with resistance to MAPK pathway inhibition: overexpression of these genes enhances transcription factor stimulating melanoma cell growth [55,56]. 

The occurrence of an alternative pathway(s) is another possible resistance mechanism. Activation of the PI3K-AKT pathway, through the loss of PTEN and BIM down-modulation, prevents melanoma cell apoptosis and stimulates cell growth, thus leading to BRAF inhibitor resistance [57]. Loss of PTEN correlates with worse PFS in patients treated with dabrafenib monotherapy [58]. Clinical trials evaluating the association of PI3K inhibitors with MAPK inhibitors are ongoing [59].

Furthermore, several pieces of evidence showed the reciprocal influence between BRAFi therapy and the immune system. On the one hand, BRAFi therapy modulates the tumor microenvironment through up-regulating the expression of melanoma antigens (e.g., MART, gp100) and peritumoral CD8+ T lymphocytes and reduction of immunosuppression cytokines and Treg [60]. On the other hand, increased expression of PD-L1 and PD-1 in tumor cells, of exhaustion markers (like TIM3 and FOXP3) on T cells, and the reduction of the frequency of CD8+ T cell infiltrate have been reported in histologic samples of patients progressing to BRAF inhibitors [61,62]. These observations were also matched with the detection of increased levels of pro-tumorigenic type 2 tumor-associated macrophages [61,62]. The combination of immune checkpoint inhibitors and target therapy might be a promising strategy to enhance immune responses and prevent immune-mediated resistance; this hypothesis is currently under evaluation in phase III clinical trials. 

Finally, given the paucity of therapeutic alternatives for metastatic melanoma patients, understanding the mechanisms of resistance to target therapies remains crucial to implement the efficacy of the current treatments and develop new strategies for a prolonged clinical benefit. 

## 3. Biomarkers in Immunotherapy

Together with target therapy, immunotherapy development, contributed to the rapid and dramatic change in melanoma’s therapeutic landscape [63]. Initially, two classes of ICIs were approved: 1. anti-CTLA-4 antibody, and 2. anti-PD-1 [64,65] antibodies, showing, when used in monotherapy, durable benefit in survival, both in BRAF mutated and wild-type patients [64].

In addition, the combination of anti-PD-1 and anti-CTLA-4 antibodies demonstrated a sharp increase in response rate and progression-free survival compared to either drug alone [66]. However, a long term clinical benefit was limited to a fraction of patients, and the significant toxicity reported in the case of combination therapy highlights the importance of the accurate selection of patients [66].

Biomarkers are still an unmet need to predict patients who will most likely benefit from different immunotherapies, those who will experience toxicities, and to identify the mechanisms to overcome the resistance to therapies (Table 2).

### 3.1. PD-1/PD-L1 Signaling

PDL-1 expression on tumor cells, assessed by immunohistochemical staining, has been extensively evaluated as a predictor of clinical response to anti-PD-1/PD-L1 therapy.

Several studies, including different tumor histology, revealed a positive correlation between PD-L1 expression and response to ICIs [64,76,77], while others did not report any significant association [78]. These contradictory results might be, in part, attributed to the lack of a clear definition of threshold and significance of PDL-1 positivity. In addition, different PD-1 and-PD-L1 specific antibodies have different complementary diagnostic tests, which also have different thresholds of positivity. In the studies with pembrolizumab, the positivity of tissues for PD-L1 is defined, using the 22C3 antibody (Merck, Co., Inc., Kenilworth, NJ, USA), as at least ≥1% positive tumor cells. For nivolumab, the 28-8 antibodies (Dako, Santa Clara, CA, USA) were used, with a threshold of positivity of at least 5% of tumor cells. This variability was reflected in the results of the clinical trials. The KEYNOTE-006 clinical trial, for instance, showed that the expression of PD-L1 was found in 80.5% of cancer cases [64], while in the Checkmate-067 study, only 23.6% of cases displayed positivity [66]. 

Moreover, while PD-L1 positivity is associated with the clinical benefit to ICIs, clinical responses could also be observed in patients with PD-L1 negative tumors. Interestingly, in clinical trials combining nivolumab and ipilimumab [66], for PD-L1 positive patients, the PFS was comparable between the two arms of single agents and the combination. In PD-L1 negative patients, the RR, PFS, and OS were significantly higher in the group treated with the combination. Altogether, these evidences highlight that the solely expression of PD-L1 might be inappropriate as predictive biomarker. The potential variability of PD-1 expression between primary and metastatic lesions [67] and its modulation over time, indicating that a single small biopsy not representative of the actual extension of the disease, represents the major limitations for the assessment of PD-L1 as a standalone biomarker [67].

On the other hand, the prognostic value of the PD-L1 expression in patients with metastatic melanoma remains even more controversial, having been associated with better or worst prognosis in different studies [79,80,81,82,83]. Indeed, the results of 2 different studies in the adjuvant setting, one comparing nivolumab to ipilimumab and pembrolizumab to placebo, respectively, showed that recurrence-free survival was longer in patients with PD-L1 positive tumors, irrespectively from the treatment [4,84]. These findings remain hypothesis generating and, further studies are required to validate the role of PD-L1 in defining patients’ prognosis and in driving clinical decisions.

### 3.2. Baseline Immune Factors

The considerable variability in intra-patient and inter-patient immunogenicity of melanoma reflects the evolutionary process between the immune system and melanoma cells, which cannot be limited to PD-1/PD-L1 interaction but must be viewed as an evolutionary process defined by a different phenotypic and functional subset of cells.

Since 2006, when Galon and co-workers demonstrated that the qualitative, the quantitative, and the spatial localization of CD3+ CD45RO+ memory T cells were independent prognostic factors for the survival in colorectal cancer (CRC) [85,86], many efforts have been placed to determine the role of immune infiltrating cells as biomarkers of immunotherapy.

Herbst et al. showed that the density of CD8+ T cells represented a reliable predictive indicator of response to PD-1 inhibition [87]. In particular, in the setting of anti-PD1/PDL1 therapy, where a possible mechanism of action is the revitalization of pre-existing T cell-mediated responses [88], the phenotype of tumor-infiltrating lymphocytes (TILs) could play a central role in predicting patients’ clinical outcome. Two different studies documented the association between the presence of effector T cells, assessed by perforin and granzyme levels [89,90], and the anti-PD-1 response was observed, highlighting the importance of the characterization of intratumoral immune cells.

In melanoma patients, PD-1 is expressed by heterogeneous populations of T cells. The use of additional T cell-associated markers, such as CTLA-1, LAG-3, TIGIT, can lead to the discrimination between an effector and exhausted cell populations. Daud et al. [70] found that TILs expressing CTLA-4 and PD-1 represent a subset of exhausted T cells associated with ICI’s clinical response.

A similar observation was performed by Huang et al., showing that the phenotype of circulating KI67+ T cells normalized to tumor burden, could be rescued toward effector cells by ICI therapy in association with a favorable clinical outcome of melanoma patients [91]. Maccalli and colleagues characterized more deeply the immunological responses in advanced melanoma patients treated with the combination of chemo-immunotherapy (fotemustine and ipilimumab). The levels of central memory T cells in the peripheral blood, expressing either co-stimulatory and activatory molecules (CD45RA− CD62L+ CCR7+ CD27+ CD28+ BTLA+/PD-1+), were associated with clinical responses [71]. 

Two different reports from Weide et al. assessed the role of 2 different baseline signatures in the peripheral blood of patients treated with ICIs. In the first one, the improved OS and PFS of metastatic melanoma patients treated with ipilimumab were associated with baseline determinations of high absolute eosinophil counts (AEC), relative lymphocyte counts (RLC), absolute monocyte counts, CD4(+)CD25(+)FoxP3(+) cells, myeloid-derived suppressor cells (MDSC) level, and low baseline LDH [72]. In the second study, the clinical benefit in patients treated with pembrolizumab was associated with low LDH and high relative lymphocyte and eosinophil counts [73].

Little attention has been given to the role of innate immunity in the context of the anti-PD1 blockade. Indeed PD-1 is expressed on natural killer (NK) cells and dendritic cells (DCs). This is particularly relevant in tumors with loss of HLA expression [92], in which tumor rejection is dependent on NK cells, and is enhanced after PD-1 blockade. Indeed, PD-1 is expressed on natural killer (NK) cells and DCs.

PD-1 blockade can also trigger or enhance the secretion of activating cytokine by tumor-infiltrating DCs, such as IL-12, IFN-γ, and CXCL9/CXCL10 [92,93]. The complexity of this information explains, at least in part, that the activity of ICIs is exploited through a series of events, with many variables influencing the impact in a heterogeneous tumor microenvironment (TME), which is rarely identical to itself. 

### 3.3. Tumor Mutational Burden

Tumors with a high frequency of non-synonymous somatic mutations, such as melanoma and lung cancer, can generate an increased number of neoantigens, resulting in ‘novel’ and highly immunogenic targets for the immune system. Importantly, this altered immunogenic molecular profile can induce potent immune responses and clinically activity of ICIs [94,95,96,97].

The tumor mutational burden (TMB) is identified as the number of non-synonymous mutations in the coding area of the tumor genome. 

In general, high TMB is associated with better survival [96], almost in most histologies [98]. Snyder et al. evaluated the presence of somatic mutations and neo-antigen load in 64 tumor samples derived from patients with melanoma treated with an anti-CTLA-4 antibody [68,99]. The mutational load was associated with the expression of neo-antigens and a subsequent correlation with the clinical response to ICIs.

Similar observations were obtained in NSCLC [100]; more recently, other studies extended these evidences to tumors with diverse histologies treated with immunotherapy. However, many challenges need to be resolved in order to use the TMB in the clinical practice as a predictive biomarker for immunotherapy. First of all, the lack of agreement on TMB assessment, the multiple platforms use for its assessment (DNA amounts, the extension of genome analyzed), and the different cut-off values render this evaluation contradictory [101,102]. 

### 3.4. Tumor Microenvironment

As described in the previous paragraphs, single factors, such as the presence of either TILs or PD-L1 in the tumor, are not sufficient to predict patients’ responsivness to immunotherapy. The lack of direct correlation mirrors the complexity of tumor-host interactions in the microenvironment, with the interplay of cancer cells with different subpopulations of immune cells, such as MDSCs, fibroblastsa, and a variety of paracrine signals. The tumor microenvironment evolves depending on both tumor-related factors, such as tumor histology and host factors, shaping the characteristics of immune infiltrate. For instance, a correlation between PD-L1 expression on immune cells (e.g., DCs, macrophages, and T lymphocytes) and response to ICIs was reported in different tumor types, including melanoma [87,103]. Additionally, there is a growing body of evidences that tumor-associated macrophages (TAM) may abrogate anti–PD-1 response in patient cohorts with advanced melanoma through different mechanisms. TAMs might weaken antitumor immune responses, probably by sequestering therapeutic anti-PD-1 antibodies from T cells [104]. Nuebert et al., demonstrated that the secretion of colony-stimulating factor-1(CSF-1) by melanoma cells upon exposure to T cell-derived cytokines recruits TAMs to the tumor site and consequently hampers antitumor immune responses [105]. Similarly, the lack of response to atezolizumab was associated with transforming growth factor β (TGFβ) signaling in tumor-associated fibroblasts, which prevent T-cell differentiation and infiltration into the tumor [106]. In this context, a possible solution to reset antitumor immune responses and to improve clinical outcomes in melanoma patients unresponsive to immunotherapy, could be the combination of immune checkpoint blockade with macrophage elimination. In conclusion, a deeper understanding of non-tumoral cells and their paracrine signaling within the TME could facilitate the discovery of biomarkers associated with the efficacy of for ICIs.

The immune infiltrate represents a multifaceted component of TME. Indeed, different preclinical and pathological studies demonstrated that the development and progression of melanoma are associated with changes in cell metabolism involving a shift from oxidative phosphorylation to aerobic glycolysis [107,108]. In BRAF-mutated melanoma, the constitutive activation of BRAF kinase triggers a metabolic rewiring which involves a cascade of consequences, including HIF-1 activation [109] which is normally induced in response to low oxygen levels and increased expression of glucose transporters and glycolytic enzymes [108] (e.g., SLC7A11). In addition, the oxygen concentration in the micro-environment depends on melanin concentration in the lesion: a retrospective analysis showed that its synthesis is related to worst disease advancement and radiotherapy outcomes [107]. 

In other preclinical works, it was shown that the inhibition of RAS/RAF pathway results in metabolic reprogramming, which can ultimately determine a decreased level of Reactive Oxygen Species (ROS) and hence a diminished antitumor effect of inhibitors of RAS/RAF pathway causing resistance to BRAF inhibition [110].

While this is still an emerging area of investigation, a growing number of preclinical works support the role of oxidative metabolism in melanoma progression and resistance to target [111] and immunotherapy [112], and a deeper understanding of metabolic alterations of cancer cells would be critical to achieving greater therapeutic success.

### 3.5. Microbiome

In the past decade, many studies have highlighted the central role of intestinal microbiota in the metabolic process and immunity [113]. Accumulated evidence indicates a crucial contribution of the microbiome also in the disease process, including carcinogenesis [114], in relationship with the establishment of a pro- or antitumor inflammatory milieu. These effects can be exploited both locally and at long distances [115]. 

For example, in CRC, the levels of *Fusobacterium nucleatum* (Fn) are increased in tumor tissues vs. normal tissues and are also increased in metastatic lesions compared to the primary tumor. Fn might mediate resistance to chemotherapy through a toll like receptor 4 (TLR4) mechanism [116]. In animal models, specific alterations in gut microbiota are associated with spontaneous antitumor immunity and different responses to CTLA-4 and PD-L1 blockade. The effect of immunotherapy with CpG oligodeoxynucleotide and anti-IL-10 mAb was dependent on a functional microbiota. 

Moreover, myeloablative regimens increase their effectiveness when they are associated with total body irradiation through augmented levels of endotoxins and, thus, of pro-inflammatory cytokines influencing the microbiome [117].

Two studies showed an association between intestinal microbiota composition and response to ICI therapy in melanoma. Gopalakrishnan et al. found that the clinical responses in 112 melanoma patients treated with anti-PD-1 therapy were associated with microbiota diversity and enriched specific subspecies of the Ruminococcaceae family [74]. This is probably because microbiota diversity is associated with an increased immune infiltrate of CD8+ T cells. Finally, the composition of the gut microbiota may also be associated with ICI-induced side effects. Dubin et al. found that in 34 patients treated with Ipilimumab, the presence of species from the Bacteroidetes phylum was associated with decreased risk of ICI-induced colitis [75]. 

The study of the human microbiota and its genetic composition (microbiome) is still in its early stages. Microbic taxa that can influence specific metabolic processes, therapeutic response to anti-cancer treatment, and related toxicity remain to be defined; nonetheless, microbiology in precision medicine will play a central role in the upcoming years.

## 4. Circulating Total DNA

Circulating total DNA (ctDNA) consists of soluble short nucleic fragments (~166 bp) released in the plasma due to cell apoptosis and necrosis [118,119]. Several studies have shown that in cancer patients, the ctDNA carries genetic information specifically present in tumor cells, providing information on cancer cells’ clonal heterogeneity and their evolution over time [118,120] (Table 3). Multiple assays can be used with different sensitivity levels to identify alterations in ctDNA. NGS-based methods may detect novel genetic aberrations or multiple co-existing mutations; in contrast, single or multiplexed locus assays identify only one or a few genetic mutations. 

Beads, emulsion, amplification and magnetics (BEAMing) and droplet digital PCR (ddPCR), two PCR-based techniques, have very high sensitivity [121,131,132,133], but they are limited by the need for a specific gene target and hence used mostly in wild type melanoma for BRAF, NRAS or c-KIT. In this subpopulation of patients, which accounts for 20% of melanoma patients, TERT promoter or TP53 might identify an additional 15% of the cases [15].

The existing knowledge, derived from ctDNA studies in melanoma, favors the demonstration that ctDNA levels function as a prognostic biomarker. Levels of ctDNA were found to significantly correlate with serological markers of disease burden, like lactate dehydrogenase (LDH), S100 calcium-binding protein B (S100B), and melanoma inhibitory activity (MIA) in melanoma specimen [134]. In addition, baseline ctDNA levels were significantly associated with tumor burden and progression-free survival (PFS) [133,134,135].

Lee et al. demonstrated that pre-operative ctDNA predicts metastasis-free survival in high-risk stage III melanoma patients undergoing complete lymph nodes dissection, independent of stage III substage136. Detectable ctDNA before complete surgical resection in patients with AJCC stage IIIB/C/D (high-risk stage III) with a BRAF, NRAS, or KIT mutant melanoma is an independent predictor of worse melanoma-specific survival (MSS) in patients receiving no systemic adjuvant therapy [122,136].

Thus, these biomarkers might be a critical tool in determining patients’ prognosis and stratifying patients for adjuvant treatment clinical trials. The size of the largest tumor-involved lymph nodes did not correlate with MSS, indicating that the prognostic significance of ctDNA detectability was not solely due to tumor volume [136]. ctDNA collected after surgery (median of two weeks after surgery) was only detectable in 12–36% of high-risk melanoma patients [122,137], but it was predictive of recurrence and survival. Median DFI was four months (95% CI 0.1–1.0) in patients with detectable ctDNA compared with 4.2 years (95% CI 2.5–limit not reached) in those where ctDNA was not detected. Sensitivity for predicting relapse was 18–55% and specificity 95%, with a positive predictive value of 79% and a negative predictive value of 51% [122,123]. The majority of patients with detectable ctDNA relapsed within one year from surgery, suggesting that ctDNA in the plasma can reveal occult metastatic disease that is not evident in radiological imaging [123].

The presence of ctDNA bearing BRAF mutation provided information regarding the responsiveness of malignant melanoma to targeted therapy that complements the usual tissue biopsy results [124,125,138,139,140]. Not only the presence but also the level of ctDNA has predictive value. In the BREAK-2 study, a phase II trial, aimed at evaluating the safety and clinical activity of the dabrafenib, showed that high basal ctDNA levels correlated with lower overall response rate and lower progression-free survival to targeted therapy [124]. These results were further confirmed in a large study that included the BREAK-3, BREAK-MB, and METRIC clinical trials [141]. Overall, these studies support the predictive value of ctDNA for the response to targeted therapy in melanoma patients.

Similar results were also observed with immunotherapy. Gray et al. showed that baseline ctDNA levels were predictive of clinical response and long term clinical benefit to anti-CTLA-4 antibodies [125]. More recently, Lee et al. reported that also in the setting of anti-PD-1 therapy, ctDNA levels at baseline provide an accurate prediction of tumor response, progression-free survival, and overall survival [139]. High levels of ctDNA can precede radiological evidence of disease progression and acquired resistance to targeted therapy. Finally, two recent studies highlighted ctDNA quantification as a suitable complementary modality to functional imaging for real-time monitoring of tumor burden [126]. Imaging integration could be useful, in particular, to differentiate pseudo-progression from real progression [136]. 

However, ctDNA is not able to detect or monitor intracranial disease activity [126]. The blood-brain barrier may restrict the release of ctDNA into the circulation. This has been shown in patients with brain metastases who had undetectable peripheral blood circulating ctDNA although the ctDNA was detectable in cerebral spinal fluid [142,143]. Finally, ctDNA might be a potential tool to monitor disease-clonal evolution. Anti-BRAF and anti-MEK therapies lead melanoma cells to a positive clonal selection, driving acquired mutation resistance. Studies have demonstrated that the identification of specific mutations of resistance, such as NRAS at codon 61 (p.Q61K/R), is associated with resistance to several drugs [125,140]. In this setting, an early therapy switches to immunotherapy, before an uncontrolled increase in tumor burden, might increase the subsequent treatment response rate. Previous studies have shown that a low tumor burden correlates with response to immunotherapy.

### 4.1. Micro RNAs 

MicroRNAs (miRNAs) are short (20–200 nucleotides) non-coding RNA molecules that regulate and modulate gene transcription processes and epigenetic processes involved in cell proliferation, differentiation, and apoptosis. miRNAs are secreted by cells into the circulation, but compared to ctDNA, they are more stable [144,145].

miRNAs have been implicated in the regulation of tumor development, progression, and metastasis, and as such, have been proposed as potential cancer biomarkers [146,147]. The expression of miRNAs has shown diagnostic, prognostic, and predictive value in melanoma [127]. Elevated levels of miRNA-221 have been observed in melanoma compared to healthy controls, and their levels correlate with the stage of the disease [148]. However, the diagnostic accuracy of miRNA can be superior when a panel of miRNAs is evaluated [128]. The major limitation of the role of miRNAs as prognostic/predictive tool is the lack of tumor-specificity [127]. Behind these favorable results, further studies are warranted to confirm the predictive and prognostic value of miRNAs in melanoma.

### 4.2. Circulating Tumor Cells

The detection of circulating tumor cells (CTC) in patients with melanoma is challenging because of the low concentration of these cells in peripheral blood and the lack of common CTC markers, as in the case of epithelial cancers (e.g., EpCAM) [149]. In addition, melanoma CTCs represent highly heterogeneous cell subpopulations [150,151], and the simultaneous usage of multiple markers is required for the isolation of CTCs. 

The variety of techniques used to identify CTCs [152,153,154] resulted in high variability of information that might limit this methodology’s current clinical application.

In some studies, monitoring the levels of CTCs before and during melanoma treatment has been shown to be informative with respect to prognosis and therapy response in melanoma [129,155]. Using real time-PCR to detect transcripts in blood, Reid and colleagues showed that in 230 patients, the presence of two transcripts (MLANA and ABCB5) was associated with disease recurrence and the expression of one other (MCAM) was significantly more common in patients with poor treatment outcomes [130]. Also, the detection of multiple melanoma markers in patients’ blood was associated with disease stage [156], disease-free survival (DFS), and overall survival (OS) [129,157]. 

Despite some encouraging results, CTCs clinical use as a reliable biomarker will remain limited because of the uncertain biology of these cells and the lack of standardized technology.

## 5. Conclusions

In the last decade, an extraordinary leap forward in the treatment of melanoma occurred, taking advantage of the advent of targeted and immunotherapies. Hence, from now on, preclinical and clinical investigations will have to face new emerging needs.

While these approaches have provided a radical improvement in RR, DFS, and OS, although a relevant proportion of the patients experienced limited clinical benefits. The “classical” clinical-histological classification of T-, N- and M-stage might no longer be appropriate in the light of new therapeutic approaches. The availability of predictive biomarkers has the potential to develop more personalized therapeutic approaches. 

For oncogene-addicted cancers, such as BRAF+ melanoma, the presence of BRAF V600E or V600K mutation represents an effective predictive tool for the choice of specific target therapies.

However, instead, for others, such as NRAS or c-KIT mutated melanoma, the mutation’s predictive power is not sufficient to identify true responders’ subpopulations.

Immunotherapy represents the only therapeutic option for wild-type melanomas and an alternative option for BRAF mutated melanoma. However, only a fraction of these patients can show clinical benefit. In this setting, despite numerous candidate biomarkers have demonstrated a correlation with clinical response, the composite nature of the factors involved in treatment efficacy requires alternative approaches for proper patient selection.

PD-L1 expression by IHC is not sufficient to stratify or to withhold patients from potentially beneficial therapy. Other characteristics may then help to select patients for ICIs treatment. These include genomic features, such as TMB, immune infiltrate, lymphocytes subpopulations, and also the composition of gut microbiota. 

The combinatorial use of these parameters might improve to define the dynamic nature of tumor/host interactions and might ultimately improve our capacity to predict response to immunotherapy.

Another unmet need in the management of melanoma patients is to develop biomarkers that predict toxicity, mostly in the case of treatments of similar efficacy or when there is the need to identify a subgroup of patients able to tolerate significant toxicities as for Nivolumab and Ipilimumab combination [158].

**Figure 1:**
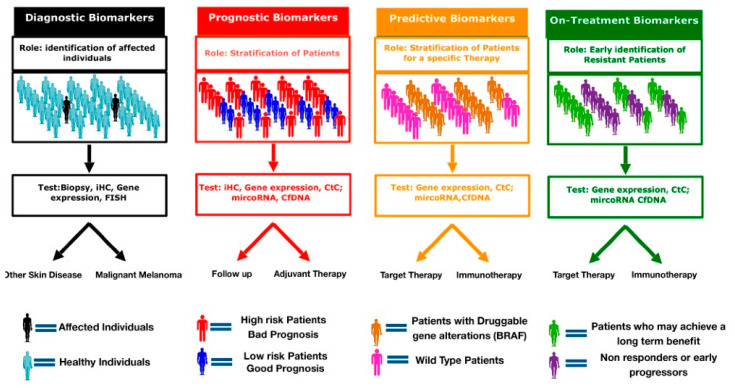
Clinical applications of cancer biomarkers. Genetic, protein and cellular components can serve as diagnostic, prognostic, predictive and/or on-treatment biomarkers. diagnostic biomarker are used identify and detect the presence of cancer in individuals. Prognostic biomarkers provide information on the risk of recurrence and expected outcomes. Predictive biomarkers forecast the potential benefit of a specific treatment. On-treatment biomarker help to identify early progressors from long responders.

Although many patients experience an initial clinical benefit to anti-melanoma therapies, acquired resistance ultimately develops. In this setting, biomarkers may provide valuable information to develop a more personalized approach. On-treatment biomarkers could provide information on early signs of resistance and allow a correct and efficient sequencing of target and immune therapy or identify patients who most likely will experience a long-term benefit (Figure 1).

To overcome the aforementioned hurdles, the clinical validation and application of biomarkers need to integrate a vast amount of information into a clinically applicable setting. This will imply systematic specimen collection and handling, the use of standardized assays available in a large number of laboratories, the development of appropriate clinical endpoints for determining early efficacy, and clinical development, integrating existing preclinical work with different platforms in development and appropriate clinical tools able to give specific answers.

## Figures and Tables

**Table 1:** Selected Biomarkers in Melanoma-Targeted Therapy.

Gene	Incidence	Comments	References
BRAF	40–60%	Correlated with response to BRAF-targeted therapies. has led to FDA approval of amplification and sequencing technologies, and multiple laboratory tests to assess BRAF mutation status	[3]
NRAS	20%	Correlated with response to MEK inhibitors	[6]
C-KIT	3% of melanoma; 20–30% melanomas arising from (CSD) skin, acral and mucosal sites	KIT-inhibitors have shown activity in patients with specific mutations	[7,8,9,10,11]
GNAQ	80% of uveal melanoma	MEK inhibitors failed to show efficacy in Phase III trials	[12]
NF1	46% of cases with BRAF and NRAS wild type	Early clinical trials	NCT02645149
PTEN	25–30%	Implicated in mechanism of resistance to MAPK inhibition	[13]
CDK2	11%	Early clinical trials	NCT02645149

**Table 2:** Selected Biomarkers in Immunotherapy.

Biomarker	Clinical Validation	Tissue for Assessment	Assay	Comments	References
PD-L1	Yes; Phase III Trial	Tumor; TME	IHC	Clinical responses in PD-L1 negative tumors. Variability of the assays	[64,67]
TMB	Yes; Phase III Trial	Blood; TME	NGS; WES	Lack of standardized TMB thresholds. Variability in quantification methods.	[68]
GEP	No; early clinical development	Tumor	IMPRES (RNA-seq)	Costs	[69]
TIL	No; early clinical development	Tumor	IF, IHC	Tumor tissue availability	[70]
Peripheral lymphocytes	No; early clinical development	Blood	IF	Role of T-cell subpopulations in predicticting clinical benefit	[71,72,73]
Gut Microbiota	No; early clinical development	Oral, gut	PCR; NGS	Inter-patients variability. Role in predicting toxicity	[74,75]

Abbreviations: TME, Tumor; Microenvironment; IHC, Immunohistochemistry; ICIs, immune checkpoint inhibitors; NGS, next-generation sequencing; WES, whole exome sequencing; GEP, gene expression profile; TMB, Tumor Mutational Burden; IMPRES, immune-predictive score; TIL, Tumor-infiltrating lymphocytes; IF, Immunofluorescence; PCR, Polymerase Chain Reaction.

**Table 3:** Circulating nucleic acids or tumor cells as biomarkers for melanoma.

Biomarker	Predictive/Prognostic	Clinical Validation	Assay	Comments	References
ctDNA	Prognostic	Advanced clinical investigation	PCR based BEAMing	ctDNA has the potential to anticipate clinical progression. Need of a specific gene target	[121,122,123]
ctDNA	Predictive	Advanced clinical investigation	PCR based	Prognostic and predictive to dabrafenib and Trametinib. Limited sensitivity for brain metastasis	[124]
ctDNA	Prognostic	Advanced clinical investigation	PCR based	Prognostic and predictive to target therapy and Immunotherapy	[125,126]
MicroRNAs	Prognostic/Predictive	Pre-clinical	Luciferase assay	miRNAs are more stable compared to ctDNA. Low Tumor specificity	[127,128]
CTC	Prognostic/Predictive	Pre-clinical	PCR based	Lack of standardized technology	[129,130]

Abbreviations: ctDNA, cellular tumor DNA; PCR, Polymerase Chain Reaction; BEAMing beads, emulsion, amplification and magnetics; miRNA, microRNA; CTC, circulating tumor cells.

## Abbreviations

Ab	Antibody
AEC	Absolute eosinophil count
AMC	Absolute monocyte count
AJCC	American Joint Committee for Cancer
AKT	Protein Kinase B
BEAMing	Beads, emulsion, amplification and magnetics
BIM	Bcl-2-like protein 11
BTLA	B-T lymphocyte attenuator
BRAF	v-Raf murine sarcoma viral oncogene homolog B1
CCR	Cytokeratin receptor
CDKN	Cyclin-Dependent Kinase Inhibitor
CDK4	Cyclin-Dependent Kinase 4
CpG	Cytosine linked to a guanine by a phosphate bond
CSD	Chronic sun damage
CSF-1	Colony-Stimulating Factor-1
CTC	Circulating tumor cells
ctDNA	Cellular tumor DNA
CTLA-4	Cyotoxic T-Lymphocyte associated protein 4
CXCL	Chemokine (C-X-C motif) ligand
DC	Dendritic cells
DFI	Disease-free survival
DNA	Desosiiribonucleic acid
ddPCR	Digital droplets polymerase chain reaction
EpCAM	Epithelial cell adhesion
Fn	*Fusobacterium nucleatum*
FOXP3	Forkhead box P3
GNAQ	G protein subunit alpha q
GNA11	Guanine nucleotide-binding protein subunit alpha-11
HLA	Human leukocyte antigen
ICIs	Immune checkpoint inhibitors
IFN	Interferon
IHC	Immunohistochemistry
IL-12	Interleukin
KIT	Proto-oncogene receptor tyrosine kinase
LAG-3	Lymphocyte-activation gene 3
LDH	Lactate dehydrogenase
MAPK	Mitogen-activated protein kinases
MDSC	Myeloid-derived suppressor cell
MET	MET proto-oncogene
MIA	Melanoma inhibitory activity
miRNA	Microrna
MITF	Melanocyte inducing transcription factor
MSS	Melanoma-specific survival
mTOR	Mammalian target of rapamycin
NF1	Neurofibromin 1
NGS	Next-generation sequencing
NK	Natural killer
NRAS	Neuroblastoma RAS Viral Oncogene Homolog
NSCLC	Non-small cell lung cancer
ORR	Overall response rate
OS	Overall survival
PD-L1	Programmed Death—Ligand 1
PCR	Polymerase chain reaction
PFS	Progression Free Survival
PI3K	Phosphatidyl inositol 3 kinases
PKC	Protein kinase c
PTEN	Phosphatase and TENsin homolog
RLC	Relative lymphocyte count
RNA	Ribonucleic acid
RTK	Receptor tyrosine kinase
TAM	Tumor-Associated Macrophages
TGFβ	Transforming Growth factor β
TIGIT	T-cell immunoreceptor with immunoglobulin and ITIM domains
TIL	Tumor-infiltrating lymphocytes
TLR	Toll-like receptor
TMB	Tumor mutational burden
TME	Tumor Microenvironment
Treg	Regulatory T-cell
WES	Whole exome sequencing
Bi	Bliography
